# Comparative Antibiotic Resistance of Diarrheal Pathogens from Vietnam and Thailand, 1996-1999

**DOI:** 10.3201/eid0802.010145

**Published:** 2002-02

**Authors:** Daniel W. Isenbarger, Charles W. Hoge, Apichai Srijan, Chittima Pitarangsi, Niyada Vithayasai, Ladaporn Bodhidatta, Kimberly W. Hickey, Phung Dac Cam

**Affiliations:** * Armed Forces Research Institute of Medical Science, Bangkok, Thailand; † Walter Reed Army Institute of Research, Silver Spring, Maryland, USA; ‡ Queen Sirikit National Institute of Child Health, Bangkok, Thailand; § Tripler Army Medical Center, Honolulu, Hawaii, USA; ¶ National Institute of Hygiene and Epidemiology, Hanoi, Vietnam

**Keywords:** drug resistance, microbial, bacteria, diarrhea, Thailand, Vietnam

## Abstract

Antimicrobial resistance rates for shigella, campylobacter, nontyphoidal salmonella, and enterotoxigenic *Escherichia col*i were compared for Vietnam and Thailand from 1996 to 1999. Resistance to trimethoprim-sulfamethoxazole, ampicillin, chloramphenicol, and tetracycline was common. Quinolone resistance remains low in both countries, except among campylobacter and salmonella organisms in Thailand. Nalidixic acid resistance among salmonellae has more than doubled since 1995 (to 21%) in Thailand but is not yet documented in Vietnam. Resistance to quinolones correlated with resistance to azithromycin in both campylobacter and salmonella in Thailand. This report describes the first identification of this correlation and its epidemiologic importance among clinical isolates. These data illustrate the growing magnitude of antibiotic resistance and important differences between countries in Southeast Asia.

Antimicrobial resistance among enteric pathogens in developing countries is a critical area of public health concern. The usual causative agents of dysentery--shigella, campylobacter, and nontyphoidal salmonella--are becoming increasingly resistant to most agents commonly in use (1–3). The current recommendation for management of travelers’ diarrhea is self-therapy with antibiotics if illness develops (4,5); fluoroquinolones are standard treatment in much of the world. Although travelers’ diarrhea has traditionally been associated with enterotoxigenic *Escherichia coli* (ETEC), some studies from Asia have shown that campylobacter is of equal or greater importance (6–8). In the early 1990s, fluoroquinolone-resistant strains of campylobacter rapidly became prevalent in Thailand, increasing from 0% to 84% of isolates from 1990 to 1995 (1).

Azithromycin has been suggested as a replacement for quinolones for empiric treatment of travelers’ diarrhea in Thailand (9). Although this drug may be a reasonable choice for travelers, its high cost precludes its use as routine treatment for dysentery in disease-endemic areas. Furthermore, the emergence in Thailand of campylobacter resistant to both quinolones and azithromycin (1) threatens the continued usefulness of both.

The seriousness of the problem has prompted calls for improved surveillance for antimicrobial resistance in developing countries, which could provide early warnings of the emergence of resistant bacterial strains (10). The prevalence of quinolone- and macrolide-resistant campylobacter in areas of Southeast Asia other than Thailand is unknown, and resistance patterns in Thailand may not apply to other countries in the region. Poorer countries such as Vietnam could benefit by avoiding a rush to newer, more expensive agents if sensitivity to older drugs could be demonstrated. Data from Vietnam relative to this issue are scarce or outdated. Surveillance data from the World Health Organization’s Western Pacific Region collaboration for 1992-93 reported 0% and 2% prevalence of fluoroquinolone resistance among nontyphoidal salmonella and shigella, respectively, in Vietnam, although the numbers of isolates and shigella groups were not specified (11). Sullivan et al. (12) reported resistance rates in >3,000 isolates of shigella, salmonella, and *Escherichia coli* collected before 1971 from U.S. soldiers and Vietnamese residents with diarrhea; levels of resistance to tetracycline, chloramphenicol, streptomycin, and novobiocem were high, particularly among shigellae. Other than these two studies, no data have been published in English on resistance patterns in enteric diarrheal pathogens in Vietnam, and no campylobacter resistance data from this country have ever been published.

The Armed Forces Research Institute of Medical Sciences in Bangkok has been coordinating studies of the causes, epidemiology, and treatment of diarrheal diseases in Thailand and Vietnam. Antibiotic susceptibility of bacterial isolates, including shigella, ETEC, and nontyphoidal salmonella from patients with diarrhea, have been tested against the most common antibiotics, including ampicillin, tetracycline, chloramphenicol, trimethoprim-sulfamethoxazole (SXT), nalidixic acid, and fluoroquinolones. In recent years, azithromycin resistance among these pathogens, as well as the emergence of fluoroquinolone and azithromycin resistance in campylobacter species, has been monitored. A review of 15 years of data from 1981 to 1995 has been published (1). Our study further documents increasing antibiotic resistance in Thailand from 1996 to 1999 and presents resistance data from Vietnam, offering insight into regional differences between two countries that are proximal geographically, but politically and economically distant.

## Methods

### Source of Isolates

Bacterial isolates from studies of community-acquired diarrhea conducted in Thailand and Vietnam from 1996 to 1999 were routinely assayed for sensitivity to various antimicrobial agents. Populations under study were predominantly children <5 years of age from both urban and rural environments but also included small numbers of adult U.S. soldiers on military maneuvers in Thailand. Some isolates were obtained from asymptomatic persons. These data archives were searched initially for all isolates of shigella (other than *Shigella dysenteriae* 1), nontyphoidal salmonella, campylobacter, and ETEC. The lists were reviewed for the presence of duplicate isolates from the same person, and duplicates were excluded.

### Microbiology

Enteric pathogens were cultured and identified by standard methods (13–15). ETEC isolates were identified with DNA probes. Antibiotic susceptibilities of shigella, salmonella, and ETEC were determined by the disk-diffusion method of Bauer and co-workers (16), with commercially prepared antibiotic disks containing ampicillin, tetracycline, chloramphenicol, sulfisoxazole, SXT, streptomycin, azithromycin, nalidixic acid, and ciprofloxacin. MIC agar-plate dilution methods (17,18) were used to test for nalidixic acid, ciprofloxacin, and azithromycin resistance among campylobacter species. Because of periodic shortages of reagents, not all isolates were tested to all antimicrobial agents.

## Results

Overall, 2,218 isolates were studied, 76% from Thailand and 24% from Vietnam. Pathogenic bacteria were obtained from subjects without gastrointestinal symptoms in 4.2%. Adult travelers (U.S. soldiers on maneuvers) accounted for 6.4% of Thai isolates, but none of the Vietnamese isolates. All other isolates were obtained from children <5 years of age. Sixty-one percent of Thai isolates were from an urban setting (Bangkok). All Vietnamese isolates were rural. All isolates were obtained either from cohorts enrolled in epidemiologic studies evaluating the prevalence of bacterial diarrhea pathogens or the incidence of diarrhea or from case-control studies evaluating diagnostic techniques.

### Routine Susceptibility Testing for Shigella Species Other than *S. dysenteriae* 1, ETEC, and Salmonella

From Thailand, 175 shigella (65% *S. sonnei*, 32% *S. flexneri*), 696 nontyphoidal salmonella, and 203 ETEC isolates were studied during the 3-year period. From Vietnam, 305 shigella (18% *S. sonnei*, 71% *S. flexneri*), 30 salmonella, and 113 ETEC isolates were studied (Table 1).

**Table 1:** Antibiotic resistance patterns among selected enteric pathogens in Thailand and Vietnam, 1996-1999^a^

		AM		CM		SXT		NA		CIP		TE		AZM	
Pathogen	Site	N	%R	N	%R	N	%R	N	%R	N	%R	N	%R	N	%R
ETEC	Thailand	203	54	203	13	203	51	203	3	203	2	203	43	68	4
	Vietnam	113	67	113	17	113	63	112	<1	113	<1	113	65	37	3
*Salmonella (all)*	Thailand	696	28	696	26	696	37	696	21	696	<1	695	59	284	5
	Vietnam	30	3	30	7	30	10	30	0	30	0	30	13	19	0
* Group B*	Thailand	258	49	258	45	258	67	258	23	258	<1	258	77	82	1
	Vietnam	6	0	6	0	6	0	6	0	6	0	6	0	3	0
* Group C*	Thailand	192	5	192	13	192	12	192	33	192	0	191	62	101	12
	Vietnam	1	0	1	0	1	0	1	0	1	0	1	0	1	0
* Group D*	Thailand	68	31	68	31	68	38	68	18	68	0	68	46	28	4
	Vietnam	7	0	7	14	7	14	7	0	7	0	7	1	4	0
* Group E*	Thailand	133	24	133	11	133	16	133	4	133	0	133	18	52	5
	Vietnam	14	0	14	0	14	7	14	0	14	0	14	7	10	0
* Other*	Thailand	45	9	45	11	45	16	45	4	45	0	45	18	21	5
	Vietnam	2	50	2	50	2	50	2	0	2	0	2	100	1	0
*Shigella (all)*	Thailand	175	29	175	21	175	90	175	<1	175	0	175	91	68	2
	Vietnam	305	77	305	64	305	78	305	<1	305	0	305	80	233	8
* Group A*	Thailand	3	0	3	0	3	0	3	0	3	0	3	33	0	NA
	Vietnam	6	50	6	17	6	50	6	0	6	0	6	50	6	0
* Group B*	Thailand	56	82	56	61	56	86	56	0	56	0	56	96	18	0
	Vietnam	216	84	216	74	216	83	216	<1	216	0	216	87	165	5
* Group C*	Thailand	2	0	2	0	2	0	2	0	2	0	2	0	0	NA
	Vietnam	28	54	28	46	28	68	28	0	28	0	28	75	22	0
* Group D*	Thailand	114	4	114	3	114	97	114	<1	114	0	114	92	50	2
	Vietnam	55	62	55	36	55	67	55	0	55	0	55	60	40	28
*Campylobacter*	Thailand	ND		ND		ND		608	73	531	77	ND		520	6
	Vietnam	ND		ND		ND		88	7	85	7	ND		62	0
C. jejuni	Thailand	ND		ND		ND		489	72	426	75	ND		415	2
	Vietnam	ND		ND		ND		71	1	69	1	ND		48	0
C. coli	Thailand	ND		ND		ND		108	75	94	80	ND		94	26
	Vietnam	ND		ND		ND		17	29	16	31	ND		14	0

^a^AM=ampicillin; CM=chloramphenicol; SXT=trimethoprim-sulfamethoxazole; NA=nalidixic acid; CIP=ciprofloxacin; TE=tetracycline; AZM=azithromycin; ND=not done; %R=percent resistant; NA=not applicable.

Among shigella, high levels of resistance to tetracycline and SXT were noted in both Thailand and Vietnam. Significantly less resistance was noted among Thai isolates to ampicillin (29% vs. 77%, p<0.05) and chloramphenicol (21% vs. 64%, p<0.05). Sorting by shigella group showed that nearly all the difference between Thai and Vietnamese isolates was accounted for by differences in the prevalence and susceptibility of *S. sonnei* compared with the more resistant *S. flexneri* strains. In Thailand, *S. sonnei* were almost all susceptible to ampicillin and chloramphenicol; in Vietnam, *S. sonnei* was more commonly resistant to these agents.

ETEC isolates demonstrated high-level resistance in both countries to ampicillin, SXT, tetracycline, and sulfisoxazole, but chloramphenicol resistance was relatively low in both countries. In contrast, nontyphoidal salmonella from Thailand had consistently and substantially higher levels of resistance to all agents, compared with those from Vietnam. Serogroup B salmonellae in Thailand were generally more resistant than other serogroups, except for serogroup C isolates, which were relatively more resistant to nalidixic acid and azithromycin.

### Quinolone and Macrolide Resistance among Shigella, Salmonella, and ETEC

Resistance to nalidixic acid was rare among shigella (<1%) and ETEC (3%) in both Thailand and Vietnam but was more common among Thai salmonella (21%). Resistance to ciprofloxacin was documented in <1% of isolates, including two salmonellae (from Thailand) and four ETEC (three from Thailand, one from Vietnam). No shigellae were resistant to ciprofloxacin in either country (Table 1).

With the exception of *S. sonnei*, azithromycin resistance was found in ≤8% of shigella, salmonella, and ETEC, with no significant difference between Vietnam and Thailand. Azithromycin resistance was present in 28% of *S. sonnei* isolates from Vietnam but only 2% of Thai isolates (p<0.05).

### Resistance of Campylobacter to Quinolones and Macrolides

We studied 608 campylobacter isolates from Thailand and 88 from Vietnam. Resistance to nalidixic acid (73% vs. 7% respectively, p<0.05) and ciprofloxacin (77% vs. 7% respectively, p<0.05) differed markedly between Thai and Vietnamese isolates. Among Vietnamese isolates, quinolone resistance was predominantly among *Campylobacter coli* rather than *C. jejuni,* but rates among Thai isolates were similar between species. Resistance to azithromycin was low in Thailand (6%), where it predominated among *C. coli*. No azithromycin resistance was present in Vietnamese isolates (Table 1).

### Co-Resistance to Quinolones and Macrolides among Campylobacters and Salmonellae

Vietnamese isolates of campylobacter were uniformly sensitive to azithromycin, so we were unable to examine correlations with quinolone resistance. Among Thai isolates, however, azithromycin resistance correlated with resistance to ciprofloxacin (p=0.007) (Table 2). Among campylobacter tested to both these agents, all isolates (n=29) that were resistant to azithromycin were also resistant to ciprofloxacin. Based on the 23% prevalence of ciprofloxacin sensitivity among azithromycin -susceptible strains, a similar rate of discordance among azithromycin-resistant strains would be expected if these resistance patterns were independent. Co-resistant isolates included both *C. coli* (n=22) and *C. jejuni* (n=7), were composed of multiple serogroups and resistance profiles, were present in both travelers and indigenous Thais, and were dispersed geographically around Thailand and temporally over the 4 years of data; therefore, they are not likely to be clonally related. These isolates were significantly more likely to be *C. coli* (76%) than were azithromycin -sensitive isolates (*C. coli* 15%; p<0.001).

**Table 2:** Co-resistance^a^ to azithromycin (AZM) and ciprofloxacin (CIP) among campylobacter isolates in Thailand, 1996-1999

		AZM

		R^a^	S
CIP	R	29^b^	358
	S	0	109

^a^R = resistant; S = sensitive. ^b^Chi square = 7.4, p=0.007.

Too few salmonellae were resistant to ciprofloxacin for analysis of cross-resistance; however, among isolates tested for resistance to both the first-generation quinolone nalidixic acid and azithromycin (Table 3), a highly significant correlation was found (p<0.001). Based on the 31% prevalence of nalidixic acid resistance among azithromycin -sensitive strains, a similar proportion would be expected among azithromycin-resistant strains if the two were independent, rather than the 80% documented.

**Table 3:** Co-resistance^a^ to azithromycin (AZM) and nalidixic acid (NA) among salmonella isolates in Thailand

		AZM

		R^a^	S
NA	R	12^b^	55
	S	3	122

^a^R = resistant; S = sensitive. ^b^Chi square = 12.5, p<.001.

### Multiple-Antibiotic Resistance among Shigellae, Salmonellae, and ETEC

Multiple resistance to ampicillin, chloramphenicol, tetracycline, SXT, and streptomycin was quite common among shigella isolates in both Thailand and Vietnam, but was significantly more so in Vietnam (46% vs. 21%, p<0.001). Lower levels of multiple resistance to these drugs were noted among salmonella and ETEC isolates from both countries (3% to 7%).

### Association between Symptoms and Prevalence of Antimicrobial Resistance

No association was found with presence or absence of gastrointestinal symptoms among isolates from Vietnam or among campylobacter or shigella isolates from Thailand. Thai ETEC and salmonellae that were isolated from participants with symptoms were significantly more likely to be resistant across the spectrum of antimicrobial agents studied (Mantel-Haensel summary chi square p=0.01 and p<0.01, respectively).

## Discussion

This study points out several new findings relative to antimicrobial resistance among enteric pathogens in Southeast Asia. We have documented the existence of quinolone resistance among campylobacter, shigella, and ETEC in Vietnam. If the pattern of campylobacter in Thailand holds true in Vietnam, one can expect rapidly increasing resistance rates to quinolones over the next few years in this genus. The reasons for the marked difference in resistance rates to this class of drugs between the two countries are unknown but likely reflect differences in human and veterinary use of fluoroquinolones.

Quinolone resistance among nontyphoidal salmonella was not seen in isolates from Vietnam, and ciprofloxacin resistance remains rare among this species in Thailand; however, progressive resistance to nalidixic acid among Thai isolates is cause for concern. Hoge (1) documented steadily increasing nalidixic acid resistance among nontyphoidal salmonellae in Thailand, from <1% in 1991-92 to 4% in 1993-94 and 9% in 1995. We documented 21% resistance in this genus during 1996 to 1999, more than double 1995 rates (chi square for trend, p<0.001). As resistance to nalidixic acid due to first-step resistance mutations is generally thought to precede resistance to fluoroquinolones (19), close continued monitoring of resistance rates to both these drugs among nontyphoidal salmonella is warranted.

Although cases of infections due to macrolide- and fluoroquinolone-resistant campylobacter have been reported, these have usually been among immunocompromised persons who have received multiple antibiotics (20–22). Our study is the first to document a statistical correlation between these two resistance patterns among a large number of community-acquired enteric isolates. Although the prevalence of azithromycin resistance remains relatively low in Thailand (6%) while ciprofloxacin resistance is high (77%), our study had sufficient power to confirm that the 100% correlation between resistance to these two agents was not likely due to chance. Previous studies of smaller numbers of azithromycin-resistant isolates from Thailand also support this 100% correlation (1,7,9). Resistance to quinolones is generally mediated by mutations in the chromosomal genes for DNA gyrase or topoisomerase IV, which are targets of action by the quinolone class, or less commonly in genes responsible for membrane flux of the drug (19), including plasmid-mediated enhanced efflux mechanisms. Resistance to macrolides most commonly results from mutations in the genes encoding ribosomal proteins but has also been associated with decreased permeability of the cell envelope in enterobacteriaceae, including plasmid-mediated mechanisms (21,23). Cross-resistance due to decreased membrane permeability has been documented between quinolones and chloramphenicol, trimethoprim, tetracycline, and cefoxitin, but not macrolides (19). Possible novel cross-resistance mechanisms in these isolates deserve to be more closely evaluated, as a common plasmid-mediated mechanism would increase the likelihood of horizontal spread.

Although resistance to quinolones among campylobacters from Vietnam was markedly lower than that from Thailand, multidrug resistance to other agents among Vietnamese shigella isolates was significantly higher than among Thai isolates, perhaps reflecting differences in human use patterns. In Vietnam in 1997, ampicillin, SXT, tetracycline, and chloramphenicol were the antimicrobial agents most commonly dispensed over the counter (24). Thai data from 10 years earlier (1987) document common dispensing of the same four drugs by Bangkok pharmacists (25), although fluoroquinolones were more frequently used for children with diarrhea admitted to Thai hospitals during 1990-1992 (26), and quinolones have likely become increasingly used in the decade since. Our data suggest that ampicillin, SXT, tetracycline, and chloramphenicol have no reasonable role in the empiric treatment of dysentery in Vietnam and should be replaced with quinolones or macrolides for this indication. Shigellae in Thailand are uniformly sensitive to quinolones, but dysentery and travelers’ diarrhea in Thailand are predominantly caused by quinolone-resistant campylobacter rather than shigella and are thus better treated empirically with macrolides. Although ampicillin resistance among Thai shigellae was stable during the 4 years of this study, data from the preceding 15 years (1) demonstrate that resistance has decreased linearly (chi square for trend, p<0.001), perhaps reflecting overall reduced human use of this drug in Thailand as other agents have become more popular.

Concern in Thailand over the possibility of increasing rates of resistance to azithromycin developing in campylobacter species is not yet borne out by our study. Hoge (1) demonstrated azithromycin resistance rates of 15% in 1994 and 7% in 1995 among campylobacters. Our data show 7% resistance among isolates from 1996 to 1999, failing to confirm an upward trend.

Limitations of our study include the fact that isolates were obtained from multiple populations, both indigenous and U.S. military, in Thailand. However, the rates documented likely reflect overall resistance among human isolates. Additionally, the populations under study in Vietnam were all from areas around Hanoi and therefore do not necessarily reflect resistance rates seen in other parts of Vietnam.

This study highlights the need to be aware of regionally specific resistance rates to avoid inappropriate antibiotic use. Additionally, given the evidence for progressive resistance to the quinolone class and co-resistance between quinolones and azithromycin among salmonellae and campylobacters, the need to develop newer classes of antibiotics to treat dysentery and travelers’ diarrhea is apparent. More efficient use of antimicrobial agents, for example, avoidance of antimicrobial therapy for acute noncholera watery diarrhea and use of effective data-based antimicrobial choices for dysentery, would likely help limit the development of resistance, although the impact of these measures would be offset by the widespread availability of antimicrobial agents over the counter in both these countries. Effective vaccines against the major causes of bacterial diarrhea are being developed but are still years away from commercial production. Parallel drug development efforts are required.
